# Lost in Translation – What is Alexithymia

**DOI:** 10.1192/j.eurpsy.2022.568

**Published:** 2022-09-01

**Authors:** A.S. Morais, R. Gomes, N. Descalço

**Affiliations:** Hospital Garcia de Orta, EPE, Serviço De Psiquiatria E Saúde Mental, Almada, Portugal

**Keywords:** alexithymia

## Abstract

**Introduction:**

Alexithymia is considered a personality trait characterized by difficulties in identifying and expressing emotions, impoverished fantasy life and tendency toward action-oriented or ‘operational’ Thinking. There are alterations in cognitive processing and regulation of emotions, and tendency to somatization.

**Objectives:**

The authors examine literature regarding the concept of alexithymia, exploring the current definition and role in the clinic, research findings and proposed management.

**Methods:**

A brief non-systematized review is presented, using the literature available on PubMed and Google Scholar.

**Results:**

Alexithymia is not a discrete psychiatric diagnosis. It has been reported in 9-10% of the general population. It is related to numerous psychiatric disorders (substance use disorders, anxiety disorders, depression and eating disorders), but also to somatic illnesses (essential hypertension, functional gastrointestinal disorders, diabetes mellitus, psoriasis, fibromyalgia and cancer pain). Neuroimaging and neurobiological studies found evidence for morphological and functional brain alterations that integrate the classification introduced by Bermond. Affective type I is characterized by the absence of emotional experience and, consequently, by the absence of cognition accompanying the emotion (associated to right unilateral cortical lesions). Cognitive Type II is characterized by a selective deficit of emotional cognition with sparing of emotional experience (associated to a right-to-left unidirectional deficit in interhemispheric transfer).

**Conclusions:**

There is little consensus on the subject. Clarification of the mechanisms underlying alexithymia can improve our management of these individuals. Identification of effective strategies could improve the patients’ capacities for adaptive emotional processing and enhance other aspects of functioning.

**Disclosure:**

No significant relationships.

